# Ginsenoside Rb1 Alleviated the AKI to CKD Transition by Targeting VEGFR2

**DOI:** 10.1111/jcmm.70732

**Published:** 2025-07-19

**Authors:** Keying Zhang, Yuwei Ji, Zhangning Fu, Xiaochen Wang, Yifan Zhang, Yan Yang, Ran Liu, Xiangmei Chen, Guangyan Cai, Quan Hong

**Affiliations:** ^1^ Department of Nephrology, First Medical Center of Chinese PLA General Hospital, State Key Laboratory of Kidney Diseases, National Clinical Research Center for Kidney Diseases, Beijing Key Laboratory of Medical Devices and Integrated Traditional Chinese and Western Drug Development for Severe Kidney Diseases Beijing Key Laboratory of Digital Intelligent TCM for the Prevention and Treatment of Pan‐Vascular Diseases, Key Disciplines of National Administration of Traditional Chinese Medicine (Zyyzdxk‐2023310) Beijing China

**Keywords:** acute kidney injury, angiogenesis, ginsenosides Rb1, vascular endothelial growth factor receptor 2

## Abstract

The decrease of peritubular capillaries, a pathological feature of acute kidney injury (AKI), is a critical part that promotes the transition of AKI to chronic kidney disease (CKD). Ginsenoside Rb1 (Rb1) has various pharmacological effects on multiple systemic diseases. However, whether Rb1 delays the transition of AKI to CKD and the mechanism are unclear. Here, we discovered that Rb1 could alleviate kidney pathological damage in mice with unilateral ischaemia/reperfusion injury (uIRI), and it enhanced kidney function, reduced renal fibrosis while increasing microvessel density. By virtual screening and molecular docking approaches, we identified vascular endothelial growth factor receptor 2 (VEGFR2) as the principal molecular target of Rb1. Furthermore, we uncovered that Rb1 activated AKT phosphorylation‐mediated angiogenesis through binding to VEGFR2, which promoted endothelial tube formation and nitric oxide (NO) release in human umbilical vein endothelial cells (HUVECs) and then relieved the endothelial dysfunction induced by hypoxia‐reoxygenation (H/R). Also, we found that Rb1 activated VEGFR2/AKT signalling in the kidney tissue of uIRI mice. Knocking down VEGFR2 or inhibiting the AKT signalling pathway can impair the proangiogenic effect of Rb1. Taken together, we demonstrated that Rb1 facilitated renal angiogenesis by activating the VEGFR2/AKT pathway of endothelial cells, thereby arresting the transition from AKI to CKD, and providing a potential therapeutic strategy for AKI.

## 1 Introduction

Acute kidney injury (AKI) is a prevalent and serious condition marked by a swift reduction in kidney function, higher occurrence rates, and quick progression [1]. The risk of chronic kidney disease (CKD) is significantly elevated by untreated or inadequately managed AKI, although it may be reversible. Epidemiological data show that AKI is a contributing factor to the onset of CKD [2]. The AKI to CKD transition has emerged as a significant public health issue, leading to considerable social and economic challenges [3].

CKD is marked by a gradual loss of the renal microvasculature, resulting in localised hypoxia and inducing profibrotic reactions. The microvasculature provides a key potential target for therapies to heal the diseased kidney [4]. The renal highly complex vascular system, including its network of glomerular and peritubular capillaries, is important for maintaining normal glomerular and tubular epithelial cell function [5]. After IRI, renal vessels fail to regenerate, leading to a 30%–50% reduction in renal blood flow, enhanced vasoconstriction, damage to renal microcirculation, and even local ischemic necrosis [6, 7]. The network of peritubular capillaries, a vital component of the renal interstitium, is key to developing renal interstitial fibrosis, frequently linked with reduced peritubular capillary density [8]. Ehling et al. [9] observed that peritubular capillary density decreased with the progression of fibrosis in different animal models. Importantly, a decrease in peritubular capillary density and progressive loss of vascular function were evident even before the onset of renal fibrosis. For a long time after the injury, reduced renal blood flow can cause hypoxia in the tubular microenvironment, leading to damage and promoting tubular interstitial fibrosis [10]. Currently, there are no effective drug therapies addressing this issue. Therefore, promoting angiogenesis during the AKI to CKD transition may represent a promising target for therapeutic intervention.

Ginsenoside Rb1 (Rb1) is a significant active component isolated from ginseng. Studies conducted recently demonstrate that Rb1 has a protective effect in various ischemia–reperfusion injuries, such as the intestine [11], heart [12], brain [13], and femoral head [14]. Earlier research has indicated that Rb1 can alleviate urinary albumin elevation and renal tissue pathology in diabetic kidney disease (DKD) and unilateral ureteral obstruction (UUO) [15, 16]. Notably, it has been demonstrated that Rb1 can mitigate CKD‐related vascular calcification by inhibiting the Wnt/β‐catenin pathway [17], suggesting that Rb1 may promote vascular repair in kidney diseases. It is still uncertain if Rb1 plays a protective role in AKI caused by uIRI and its progression to CKD, along with the mechanisms involved.

Current research indicates that protecting the peritubular capillaries could be an effective therapeutic approach for AKI induced by I/R injury. Exogenous vascular endothelial growth factor (VEGF) treatment has manifested a therapeutic impact in the early stage of AKI via increasing capillary density and inhibiting neutrophil infiltration [18]. In addition, salvianolic acid A has been demonstrated to protect against ischaemia–reperfusion AKI in rats by alleviating the damage to peritubular capillaries and endothelial cells [19]. However, there are few studies on treating AKI to CKD transition by small molecule compounds promoting angiogenesis. In our study, the effect of Rb1 on the AKI to CKD transition period was observed, and its pharmacological mechanism was investigated via virtual screening, molecular docking, and other methods. These findings may contribute to the exploration of drug targets and efficacious therapies for the AKI to CKD transition.

## 2 Methods and Materials

### 2.1 Mouse Model and Experimental Design

Male C57BL/6J mice (6–8 weeks of age, 16–22 g), purchased from Speifu Laboratory Animal Corporation, were reared in a specific pathogen‐free facility in the Animal Centre of the People's Liberation Army General Hospital of China. A model of unilateral ischaemia/reperfusion injury (IRI) was established by us for the experiment. As recently described, uIRI surgery was performed [20]. To summarise, after administering pentobarbital (40 mg/kg, i.p.) for anaesthesia, the left renal pedicle was clamped for 28 min after incision on the left side to achieve ischaemia. The animal's body temperature was maintained at 37°C during the operation. After 7 days of operation, mice received oral gavage treatment with Rb1 (112127, J&K Scientific, Beijing, China) at a daily dose of 40 mg/kg body weight for a week.

The Institutional Animal Care and Use Committee of the Chinese People's Liberation Army General Hospital rigorously reviewed and endorsed the protocols employed for handling all animals.

### 2.2 Renal Function Analysis

The specific operation was performed as described previously [21]. The transdermal glomerular filtration ratio (GFR) technique (MediBeacon GmBH, Mannheim, Germany) was used to measure awake mice's GFR. Before the experiment, the MediBeacon transdermal Mini GFR monitor was applied to the exposed skin. Additionally, each mouse received an intravenous injection of Fluorescein isothiocyanate (FITC)‐sinistrin, dosed at 70 mg per kilogram of body weight. The mice were placed in a cage, and the fluorescence signal was detected for 2 h. Then, the chip data are read. Data were analysed using MB Studio software (MediBeacon GmBH, Mannheim, Germany). The levels of blood urea nitrogen and creatinine were measured using the respective assay kits: the urea assay kit (Nanjing JianCheng, China) and the creatinine (Cr) assay kit (Nanjing JianCheng, China).

### 2.3 Kidney Histology

Kidney tissues were fixed in formalin, then embedded in paraffin and sectioned to a thickness of 2–5 μm. After dewaxing, the slices were stained with periodic acid‐Schiff (PAS), Masson's Trichrome Stain (MASSON) and Sirius red staining. Image J software (NIH, Bethesda, USA) was utilised to analyse digitised images acquired through microscopic inspections (Zeiss Spot, Carl Zeiss, Göttingen, Germany).

The severity of tubular damage was determined by evaluating sections stained with PAS, which was carried out in a blinded manner. The characterisation of acute tubular necrosis (ATN) was based on a scoring system that considered the degree of tubular necrosis, the occurrence of cast formation, the dilation of tubules and the disappearance of brush borders. Ten nonoverlapping fields (200×) were randomly selected at the cortical medullary junction area and scored from 0 to 4: 0: normal; 1: mild to moderate injury, ≤ 25%; 2: severe injury, 26%–49%; 3: high severe injury, 50%–75%; and 4: extensive injury, > 76%.

### 2.4 Western Blot

Renal tissue was lysed in RIPA buffer (Solarbio, Shanghai, China) at 4°C for 30 min. Subsequently, the sample was centrifuged at 4°C, 15,000 rpm for 30 min, and then the supernatant was collected. Following the manufacturer's guidelines, the protein concentration was determined using the BCA protein analysis kit (Genstar, Beijing, China). The protein was separated by 10% SDS‐PAGE and then transferred to a polyvinylidene difluoride (PVDF) membrane (Millipore, Darmstadt, Germany). After blocking with 5% BSA for 2 h, the PVDF membrane was incubated overnight with primary antibody at 4°C and then with HRP‐conjugated secondary antibody at room temperature for 2 h. The primary antibodies used are as follows: Collagen I (1:1000, ab254113, Abcam, the UK); Vimentin (1:1000, ab92547, Abcam, the UK); eNOS (1:1000, sc376751, Santa Cruz, USA) p‐VEGFR2 (1:1000, 2478s, Cell Signalling Technology, USA); VEGFR2 (1:1000, ab315238, Abcam, the UK); p‐AKT (1:1000, 4060, Cell Signalling Technology, USA); AKT (1:1000, 9272s, Cell Signalling Technology, USA); β‐actin (1:10,000, 66009‐1‐Ig, proteintech, China); Vinculin (1:10,000, 66305‐1, proteintech, China). Images were analysed using ImageJ software.

### 2.5 Immunofluorescence Staining

Immunofluorescence staining (IF) was performed as described previously [22]. Kidney tissue sections were blocked with 5% BSA for 60 min, incubated with CD31 (1:50, SC‐376764, Santa Cruz, USA) for 16–18 h at 4°C, and then incubated with Cy3‐conjugated secondary antibodies (red) for 2 h at room temperature. Tissue sections were imaged through confocal fluorescence microscopy to visualise fluorescence staining. Regarding capillary intensity, the percentage of CD31 area (red staining) was quantified using the Image J image analysis system.

### 2.6 Immunohistochemical Staining

Immunohistochemical staining (IHC) was performed as described previously [22]. Following the standard procedure, 3‐μm‐thick paraffin‐embedded sections were first deparaffinised and hydrated. Endogenous peroxidase was inactivated with 3% H_2_O_2_ for 30 min. The sections underwent antigen retrieval in a microwave for a period of 10 min and were incubated with primary antibody overnight at 4°C. The following primary antibodies were used: Collagen I (1:800, ab270993, Abcam, the UK); Vimentin (1:200, ab92547, Abcam, the UK) and endomucin (1:200, sc‐65495, Santa Cruz, USA). After the addition of the secondary antibody, incubation was carried out at ambient temperature for 40 min. DAB colour development was monitored under a microscope, and haematoxylin counterstaining was performed before sealing the sample with neutral adhesive. Randomly, six fields from the renal cortex within each section were selected for examination under a light microscope at a 200‐fold magnification. Cytoplasmic or cytoplasmic membrane staining in a yellowish‐brown hue indicated positive expression. ImageJ software was used to analyse the proportion of the positive area (positive area/total area).

### 2.7 Target Prediction of Angiogenesis

In the OMIM database (https://www.omim.org/) and GeneCards database (https://www.genecards.org/), we set the keyword “angiogenesis”, retrieved the gene expression profiles associated with angiogenesis, and set “Organism” to “*Homo sapiens*” to find a dataset that fits the criteria.

### 2.8 Virtual Screening and Molecular Docking

Firstly, obtain the structure of the target protein. The structure of VEGFR2 is downloaded (PDB ID: 1ywn) from the RCSB PDB database (http://www.rcsb.org/). Then, a small molecule compound library is selected. The compound library, the traditional Chinese medicine monomer compound library (HY‐L065), is provided by MedChemExpress (MCE, Shanghai, China), and a total of 2932 compounds are screened. The three‐dimensional structure diagram of Rb1 (Compound CID: 9898279) can be obtained from the PubChem database as the ligand file for subsequent molecular docking. For molecular docking, use AutoDock Vina (version 1.1.2) to optimise the protein and all molecules, including hydrogen addition, charge addition, and structural and force field optimisation. Specific operation was performed as described previously [23].

### 2.9 GO and KEGG Pathway Enrichment Analysis

The GO database (http://www.geneontology.org) consolidates data from biological model databases and research, enabling the analysis of genetic information related to cellular components (CC), molecular functions (MF), and biological processes (BP). The Kyoto Encyclopedia of Genes and Genomes database (KEGG, https://www.genome.jp/kegg/) is a database for systematic gene function analysis, including the latest gene function annotations. The targets were imported into the DAVID database (https://david.ncifcrf.gov/) for GO and KEGG analysis, and the relevant data of CC, MF, BP, and KEGG pathways were obtained. Each analysis result was ranked according to the smallest to most significant *p* value, and a *p* value ≤ 0.05 was used as the screening condition. The top 20 results of GO enrichment analysis and KEGG pathways were selected.

### 2.10 Nitric Oxide (NO) Measurements

Mice's serum and cell supernatants were collected. The NO concentration was quantified using a NO assay kit (Nanjing Jiancheng, A012‐1‐2), according to the manufacturer's instructions.

### 2.11 Cell Culture

Human umbilical vein endothelial cells (HUVEC) (from the ATCC) were cultured in RPMI medium 1640 basic 1X (Gibco, 6123092, China) containing 10% FBS, 1% penicillin, and streptomycin in an atmosphere of 5% CO_2_ and 95% air at 37°C. The cell hypoxia/reoxygenation (H/R) model was established as follows. Briefly, HUVECs were exposed to hypoxic conditions (37°C, 1% O_2_, 94% N_2_, and 5% CO_2_) for 12 h in the medium, without glucose and serum, to induce hypoxic injury. Subsequently, the medium was replaced, and the cells were cultured under normal conditions (37°C, 5% CO_2_, 95% air) for reoxygenation for 12 h, according to the experimental design. The control group was cultured under normal conditions (5% CO_2_ and 95% air) [24, 25].

VEGFR2 siRNA was purchased from GenePharma (Shanghai, China) for siRNA knockdown. Following the manufacturer's instructions, cell transfection was performed using GP‐transfect‐Mate (Suzhou, Jiangsu, China). To inhibit the activation of the AKT signalling pathway, we treated cells with the AKT inhibitor MK‐2206 2HCl (S1078, Selleck, the USA).

The siRNA target sequence is as follows. VEGFR2: 5′‐GCAUCAGAAUAAGAAACUUTT‐3′, 5′‐AAGUUUCUUACGCUGAUGCTT‐3′; Negative Control: 5′‐UGACCUCAACUACAUUGGUUTT‐3′, 5′‐AACCAUGUAGUUGAGGUCATT‐3′.

### 2.12 Cell Viability Assay

Cell viability was conducted with the Cell Counting Kit‐8 (CCK‐8) (CK‐04, Dojindo, Kumamoto, Japan). HUVECs were treated with various concentrations of Rb1 solution (concentration: 0, 0.1, 1, 10, 100 μM). The control group of cells was cultured with a medium with 0.1% DMSO. Rb1 was added during hypoxia, and the normal medium was changed during reoxygenation. The plates were incubated under anoxic conditions for 12–24 h, respectively, and then reoxygenated for 12 h.

### 2.13 Quantitative Real‐Time PCR (qRT‐PCR)

RNA was extracted from kidney samples or cells using Trizol reagent (Invitrogen, Carlsbad, CA, USA), and then cDNA was produced with the ProtoScript II First Strand cDNA Synthesis Kit (6215A, TAKARA, Japan). qRT‐PCR analyses were run on the Applied Biosystems 7500 platform (Applied Biosystems, Foster City, CA, USA), with SYBR Green Mastermix for gene expression quantification (4367659, ThermoFisher, Germany). The primer sequence for qRT‐PCR is as follows: VEGFR2: forward primer: 5′‐AGGGAGTCTGTGGCATCTGAAGG‐3′; and reverse primer: 5′‐GTGGTGTCTGTGTCATCGGAGTG‐3′; GAPDH: forward primer: 5′‐TGCACCACAACTGCTTAGC‐3′; and reverse primer: 5′‐GGCATGGACTGTGGTCATGAG‐3′.

### 2.14 Tube‐Formation Assay

A tube‐formation assay was performed to examine the proangiogenic effects of the Rb1. HUVECs (2–4 × 10^4^) were cultured in a 96‐well plate (Corning) coated with 50 μL of Matrigel Basement Membrane Matrix (Corning, 356230, USA) and incubated at 37°C for 4–6 h. Tubes were observed and measured in microscopic fields. The tube formation was quantitatively analysed with ImageJ software. ImageJ was used to analyse the tube formation ability's junctions, meshes, branches, and length.

### 2.15 Statistical Analyses

All data were presented as mean ± standard deviation (SD). Statistical analysis was performed using GraphPad Prism 8.0 (GraphPad Software, San Diego, CA, USA). Student's *t* test was applied for two‐group comparisons and for multiple group comparisons. A two‐sided *p* value < 0.05 was considered statistically significant.

## 3 Result

### 3.1 Rb1 Improved Renal Function and Alleviated Pathological Damage in the AKI to CKD Transition

In the experiment, the concentration and method significantly impacted the therapeutic effect of Rb1. Research reports have mentioned that two doses (50 mg/kg, 70 mg/kg) of Rb1 can alleviate renal fibrosis in UUO mice [16]. Furthermore, the research by Zhang et al. pointed out that a medium dose (40 mg/kg) of Rb1 could improve drug‐toxic AKI [26]. In our previous study, STZ‐induced DKD mice were treated with 40 mg/kg of Rb1 by gavage, which showed a good therapeutic effect [15]. Based on these research results, in this study, a medium dose (40 mg/kg·d) was selected, and AKI model mice were treated by gavage administration.

**FIGURE 1:**
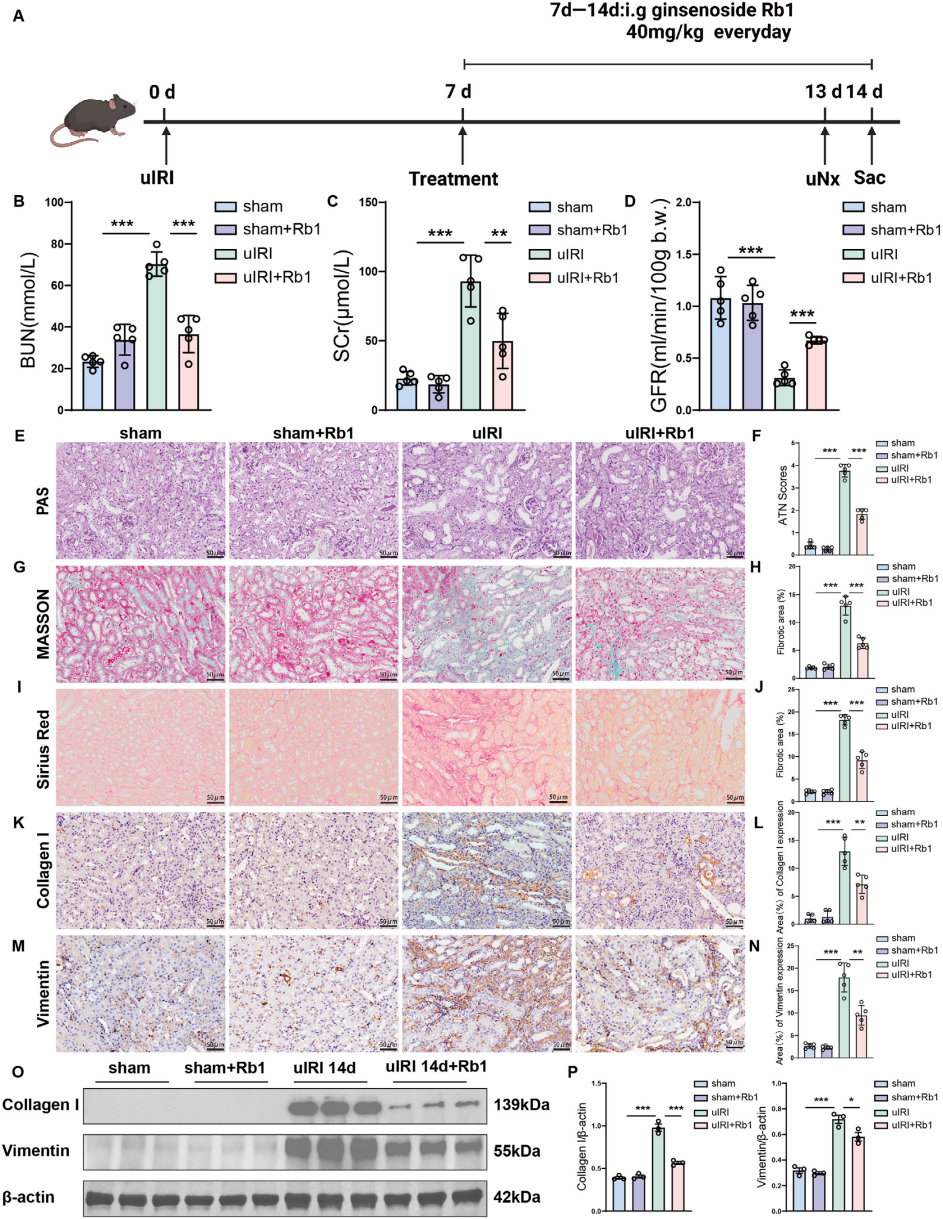
Rb1 improved renal function and alleviated pathological damage in the AKI to CKD transition. (A) Diagram showing the experimental design. Arrows indicate the time points undergoing uIRI, treatment, uni‐nephrectomy (uNx) and sacrifice (Sac). (B) Blood urea nitrogen after uIRI. (C) Serum creatinine after uIRI. (D) GFR after uIRI. (E, F) Analysis of representative images and histological features of PAS staining in renal tissue sections of different groups. Scale bar: 50 μm. (G, H) Representative images and quantitative analysis of Masson staining in renal tissue sections of different groups. Scale bar: 50 μm. (I, J) Representative images and quantitative analysis of Sirius red staining in renal tissue sections of different groups. Scale bar: 50 μm. (K, L) IHC and quantitative data of collagen I in renal tissues of different groups. Scale bar: 50 μm. (M, N) IHC and quantitative data of vimentin in renal tissues of different groups. Scale bar: 50 μm. (O, P) Western blot analysis was performed to detect the expression of collagen I and vimentin, and representative images and quantitative results were displayed. Data are presented as the means ± SD. *n* = 3 or 5 mice per group, ***p < 0.001, **p < 0.01, *p < 0.05 versus sham group. ***p < 0.001, **p < 0.01, *p < 0.05 versus uIRI group.

We constructed the uIRI model to investigate the pathological changes in the kidney during the AKI to CKD transition treated with Rb1. Renal function and pathology were evaluated at 14 days post‐uIRI (Figure 1A). After 14 days of uIRI, the mice's GFR was significantly reduced when compared to the sham mice. However, Rb1 administration significantly increased the GFR in uIRI mice. Also, serum creatinine (SCr) and blood urea nitrogen (BUN) levels were elevated in the uIRI group but significantly decreased in mice treated with Rb1 (Figure 1B–D). Histopathological analysis revealed that acute tubular necrosis (ATN) scores were lower in the uIRI + Rb1 group compared to the uIRI group (Figure 1E,F). We examined renal fibrosis changes and found an increase in collagen fibre deposition in the uIRI group, as shown by Masson staining and Sirius red staining, which was attenuated in the treatment group (Figure 1 G‐J). Results from IHC and Western blot analyses revealed a significant upregulation of Vimentin and Collagen I in the uIRI animal model, as depicted in Figure 1K–N. However, a substantial decrease in these protein expressions was observed following Rb1 administration, as illustrated in Figure 1O,P.

### 3.2 Rb1 Improved Angiogenesis and Reduced Injury Reduced by H/R In Vitro

**FIGURE 2:**
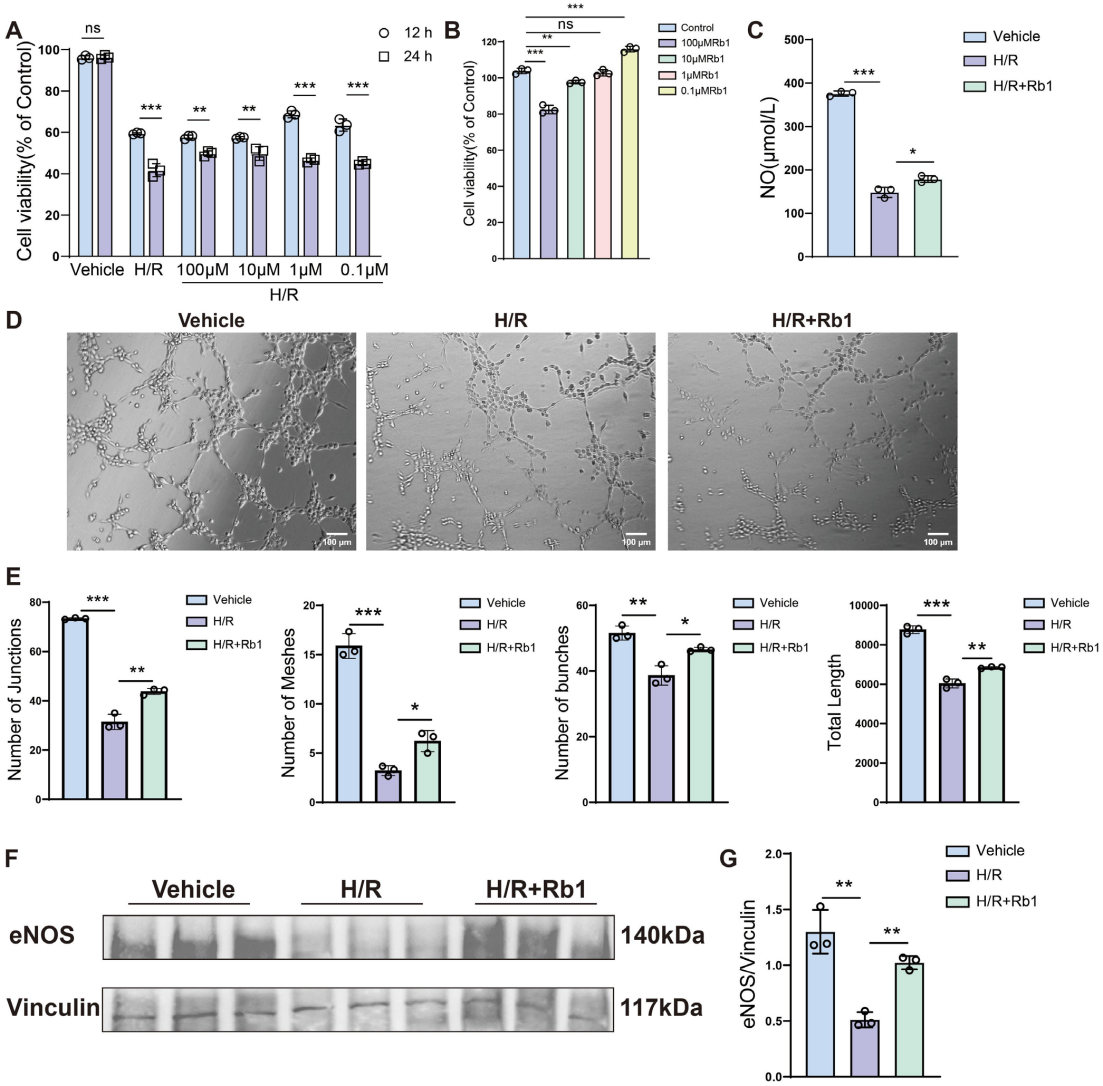
Rb1 improved angiogenesis and reduced injury caused by H/R in vitro. (A) Viability of HUVECs after Rb1 treatment under hypoxic and reoxygenation conditions. Vehicle is the control group in the cell experiment. (B) Viability of HUVECs after Rb1 treatment under normal conditions. (C) Measurement and quantitative analysis of NO in the cell supernatant. (D) Representative images of the tube formation ability after stimulating cells with Rb1. Scale bar: 100 μm. (E) Quantitatively analyse the effect of Rb1 on the formation of HUVECs tubular structure. (F, G) Representative western blot images and quantitative analysis of eNOS in kidney tissues. Data are presented as the means ± SD. *n* = 3 pre groups of cells, ***p < 0.001, **p < 0.01, *p < 0.05 versus sham group. ***p < 0.001, **p < 0.01, *p < 0.05 versus uIRI group.

To detect the effect of Rb1 on HUVECs cell viability, we used the CCK‐8 assay. The vehicle is the group with the drug solvent DMSO added. Initially, we examined the cell viability of HUVECs treated with Rb1 at concentrations ranging from 0.1 to 100 μM under hypoxic conditions for 12 h and 24 h, respectively (Figure 2A). Next, we examined the cell viability of HUVECs treated with Rb1 at various concentrations under normal conditions. We found that Rb1 at a concentration of 1 μM had no toxic effect on cells under normal conditions (Figure 2B). Our results showed that Rb1 at 1 μM significantly promoted HUVEC viability after 12 h of hypoxia. Endothelial nitric oxide synthase (eNOS) is mainly expressed in endothelial cells. It maintains blood vessel dilation, controls blood pressure, and has many other vascular protective and anti‐atherosclerotic effects [27]. The mediator NO produced by eNOS is an important part of vascular homeostasis [28]. NO measurement indicated that H/R decreased NO levels in the cell supernatant. After treatment with Rb1, NO levels increased (Figure 2C). Rb1 improved the tube‐forming ability compared with the model of H/R‐induced HUVECs, significantly increasing the number of branches, meshes, junctions and total length formed by HUVECs (Figure 2D,E). Data revealed that eNOS in the model of H/R‐induced HUVECs was significantly lower. However, Rb1 effectively increased eNOS protein expression (Figure 2F,G). In summary, our findings demonstrate that Rb1 is critical in promoting angiogenesis and enhancing vascular function.

### 3.3 Rb1 Promoted Angiogenesis and Improved Vascular Function During the AKI to CKD Transition

**FIGURE 3:**
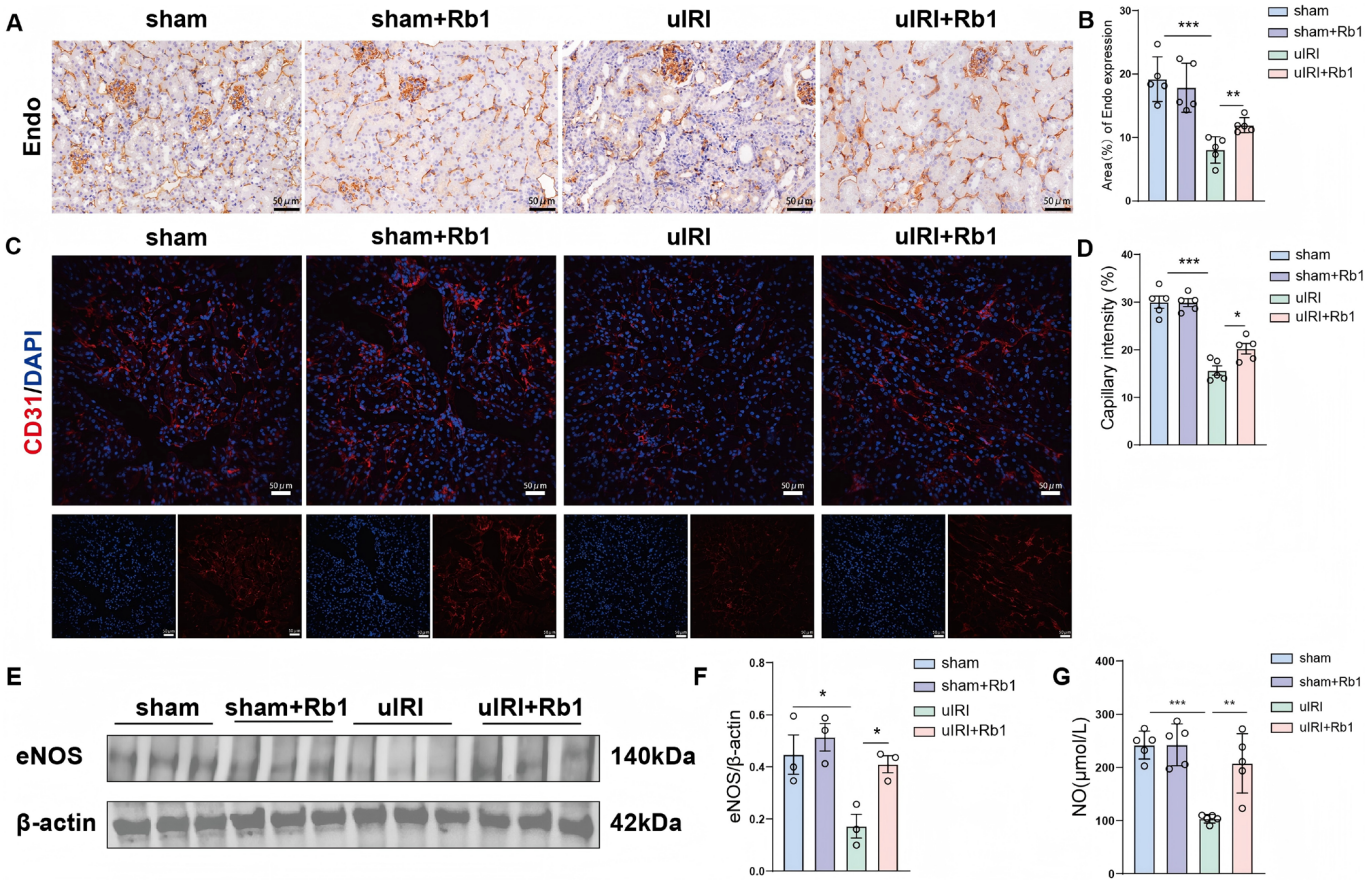
Rb1 promoted angiogenesis and improved vascular function during the AKI to CKD transition. (A, B) Representative images and quantitative results of Endo immunocytochemistry in renal tissues of mice. Scale bar: 50 μm. (C, D) Representative images and quantitative results of CD31 immunofluorescence staining in renal tissues of mice. Scale bar: 50 μm. (E, F) Western blot analysis was performed to detect the expression of eNOS and to display their representative images and quantitative results. (G) The NO measurement of serum and the quantitative analysis. Data are presented as the means ± SD. *n* = 5 mice per group, ***p < 0.001, **p < 0.01, *p < 0.05 versus sham group. ***p < 0.001, **p < 0.01, *p < 0.05 versus uIRI group.

Endomucin is mainly expressed by endothelial cells in microcapillary venules and is found in highly vascularised tissues such as the kidney and lung [29]. CD31 is typically located in vascular endothelial cells. It can engage in physiological activities like cell migration and angiogenesis and is a commonly utilised marker for endothelial differentiation [30]. To evaluate peritubular capillary density, we assessed endomucin and CD31 expression. No significant difference in blood vessel density was observed between the sham mice and the sham+Rb1 mice. The results showed that peritubular capillary density of kidney decreased in the uIRI mice, whereas it increased in the uIRI+Rb1 mice, suggesting that Rb1 treatment promotes angiogenesis (Figure 3A–D). Western blot results proved that eNOS expression was decreased in the uIRI mice, but this reduction was reversed by Rb1 treatment (Figure 3E,F). NO measurement results indicated the levels of NO in the animal serum. The expression level of NO was diminished in uIRI mice yet elevated in uIRI+Rb1 mice (Figure 3G). Collectively, these data suggest that Rb1 may improve vascular function.

### 3.4 VEGFR2 Is the Main Target of Rb1

**FIGURE 4:**
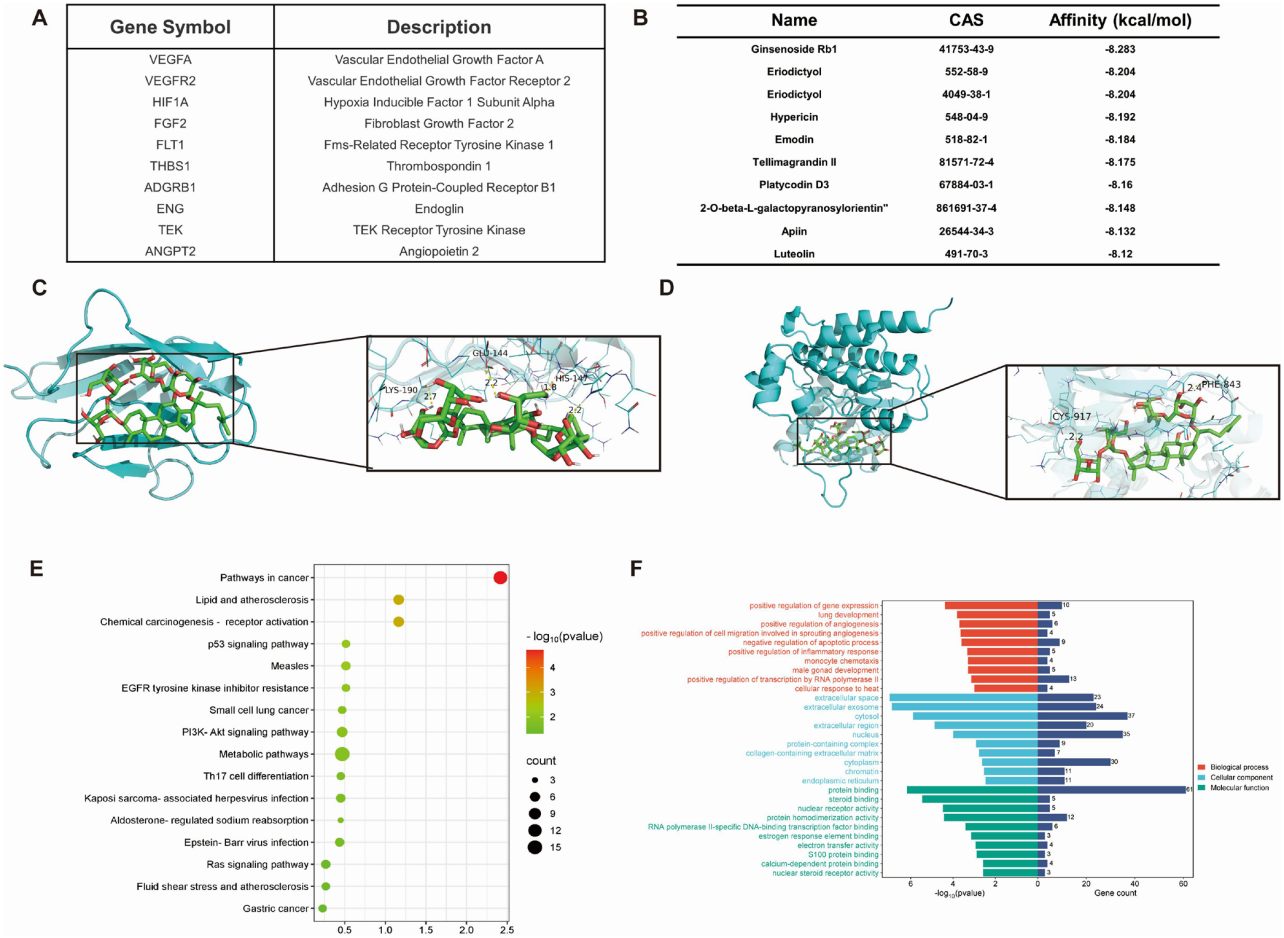
VEGFR2 is the main target of Rb1. (A) Target screening for angiogenesis. (B) The results of virtual screening. (C, D) Molecular docking simulation showed that Rb1 interacts with VEGFR1 and VEGFR2. (E) Enrichment analysis of KEGG pathway of the common target between Rb1 and angiogenesis. (F) GO enrichment analysis showed the common target of Rb1 and angiogenesis.

We can screen for drug and disease targets through major databases to further analyse drugs, diseases, targets, and pathways [31, 32]. Using “angiogenesis” as the search term, we identified 200 targets from the OMIM database and 6136 targets from the GeneCards database. On the basis of their relevance score, 10 targets associated with angiogenesis were selected (Figure 4A). Virtual screening, which rapidly evaluates large compound libraries using algorithms such as molecular docking, pharmacophore modelling, and other screening models, is an effective method for discovering lead compounds from natural product libraries [33]. We used virtual screening to evaluate 2932 compounds from the traditional Chinese medicine monomer compound library. We found that Rb1 binds well to VEGFR2 (Figure 4B). Molecular docking confirmed that Rb1 binds to both VEGFR1 and VEGFR2 with binding affinities of −4.0 kcal/mol and −7.5 kcal/mol. The binding models and energies of VEGFR1, VEGFR2 and Rb1 are shown in Figure 4C,D. VEGFRs primarily include VEGFR1, VEGFR2 and VEGFR3. VEGFR1 and VEGFR2 both promote angiogenesis, whereas VEGFR3 regulates lymphangiogenesis. VEGFR2 is more active than VEGFR1 and is a major receptor that promotes angiogenesis [34]. Therefore, we chose VEGFR2 as our primary target. Using the DAVID website to screen for common targets between Rb1 and angiogenesis, GO and KEGG enrichment analyses were conducted and identified signalling pathways with *p* < 0.05. The GO results revealed 10 molecular functions (MF), 11 biological processes (BP), and 10 cell components (CC). The KEGG analysis indicated involvement in 14 pathways, including the PI3K/AKT and EGFR pathways. Among them, the AKT signalling pathway represents the downstream pathway of VEGFR2. Upon activation, VEGFR2 can activate the AKT signalling pathway, essential for promoting angiogenesis [35]. KEGG and GO results demonstrated that Rb1 might activate the AKT signalling pathway via VEGFR2 (Figure 4E,F).

### 3.5 Rb1 Activates the Downstream AKT Signalling Pathway by Targeting VEGFR2

**FIGURE 5:**
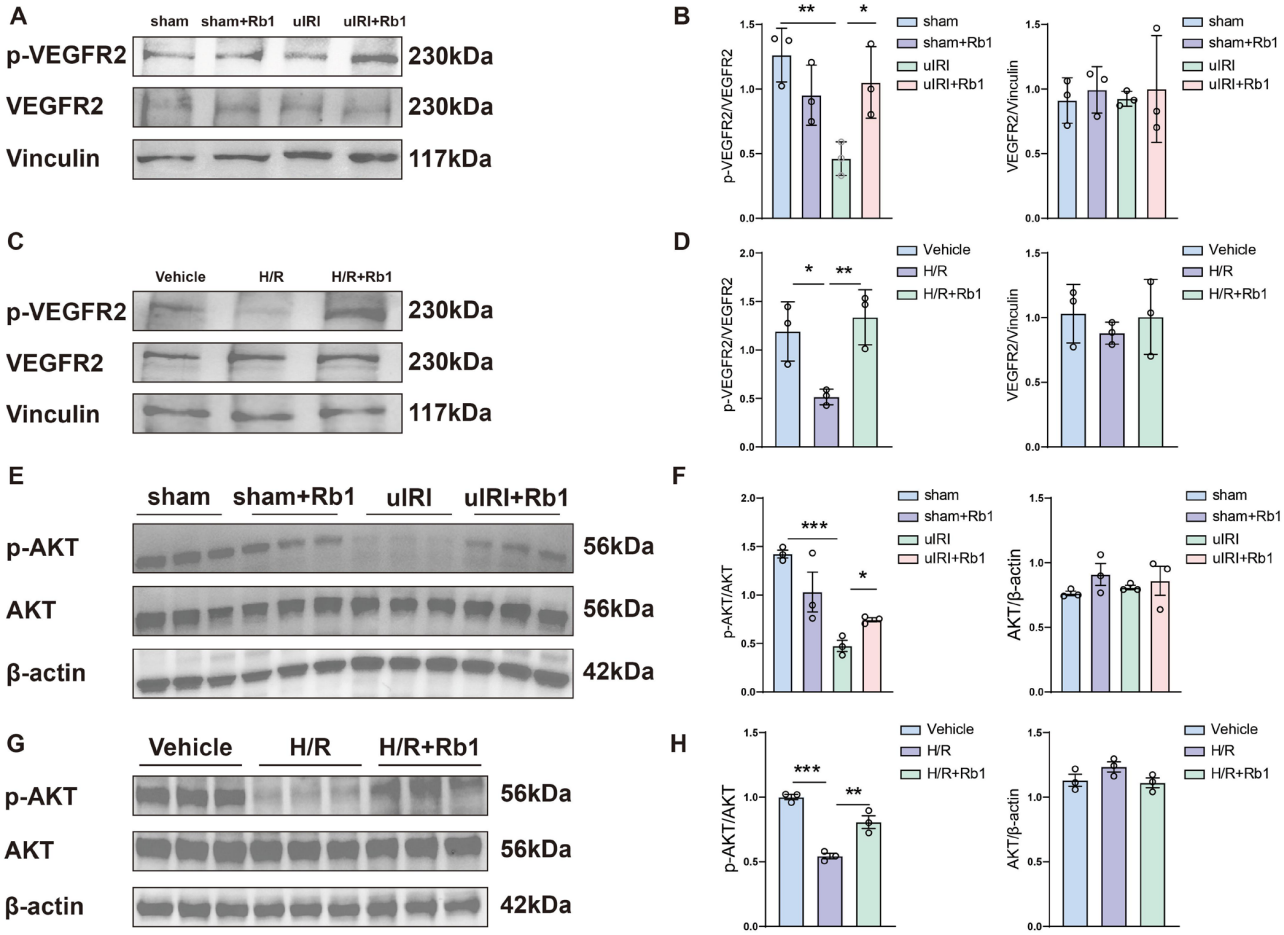
Rb1 activates the downstream AKT signalling pathway by binding to VEGFR2. (A, B) Representative western blot images and quantitative analysis of p‐VEGFR2 and VEGFR2 in mice. (C, D) Representative western blot images and quantitative analysis of p‐VEGFR2 and VEGFR2 in HUVECs. (E, F) Representative western blot images and quantitative analysis of p‐AKT and AKT in mice. (G, H) Representative western blot images and quantitative analysis of p‐AKT and AKT in HUVECs. Data are presented as the means ± SD. *n* = 3 mice or groups of cells, ***p < 0.001, **p < 0.01, *p < 0.05 versus sham group. ***p < 0.001, **p < 0.01, *p < 0.05 versus uIRI group.

As supported by previous studies, VEGFR2 can promote angiogenesis through the AKT signalling pathway [36, 37]. To verify whether Rb1 activates the AKT pathway through VEGFR2, we used western blot to detect the expression levels of phosphorylated VEGFR2 (p‐VEGFR2) and phosphorylated AKT (p‐AKT). The results showed that in kidney tissue from uIRI mice, the expression of p‐VEGFR2 and p‐AKT was significantly lower than in sham mice. In contrast, the expression of both p‐VEGFR2 and p‐AKT increased after Rb1 treatment (Figure 5A,B). In cell experiments, the expression of p‐VEGFR2 in the H/R‐induced HUVECs model was lower than in the Vehicle model. After Rb1 treatment, the expression of p‐VEGFR2 in HUVEC increased (Figure 5C,D). Previous studies have shown that Rb1 can promote angiogenesis and endothelial function by activating the AKT signalling pathway [38, 39]. To further confirm whether Rb1 activates the AKT pathway via VEGFR2, we detected the protein levels of p‐AKT and total AKT by using western blot. The results indicated that p‐AKT expression decreased in the kidney of uIRI mice, whereas it increased after Rb1 treatment (Figure 5E,F). Similarly, in cell experiments, p‐AKT protein expression significantly decreased after H/R. We observed that Rb1 increased H/R‐induced phosphorylation of AKT in HUVECs (Figure 5G,H).

### 3.6 Inhibition of the VEGFR2/AKT Signalling Pathway Reverses the Therapeutic Effects of Rb1

**FIGURE 6:**
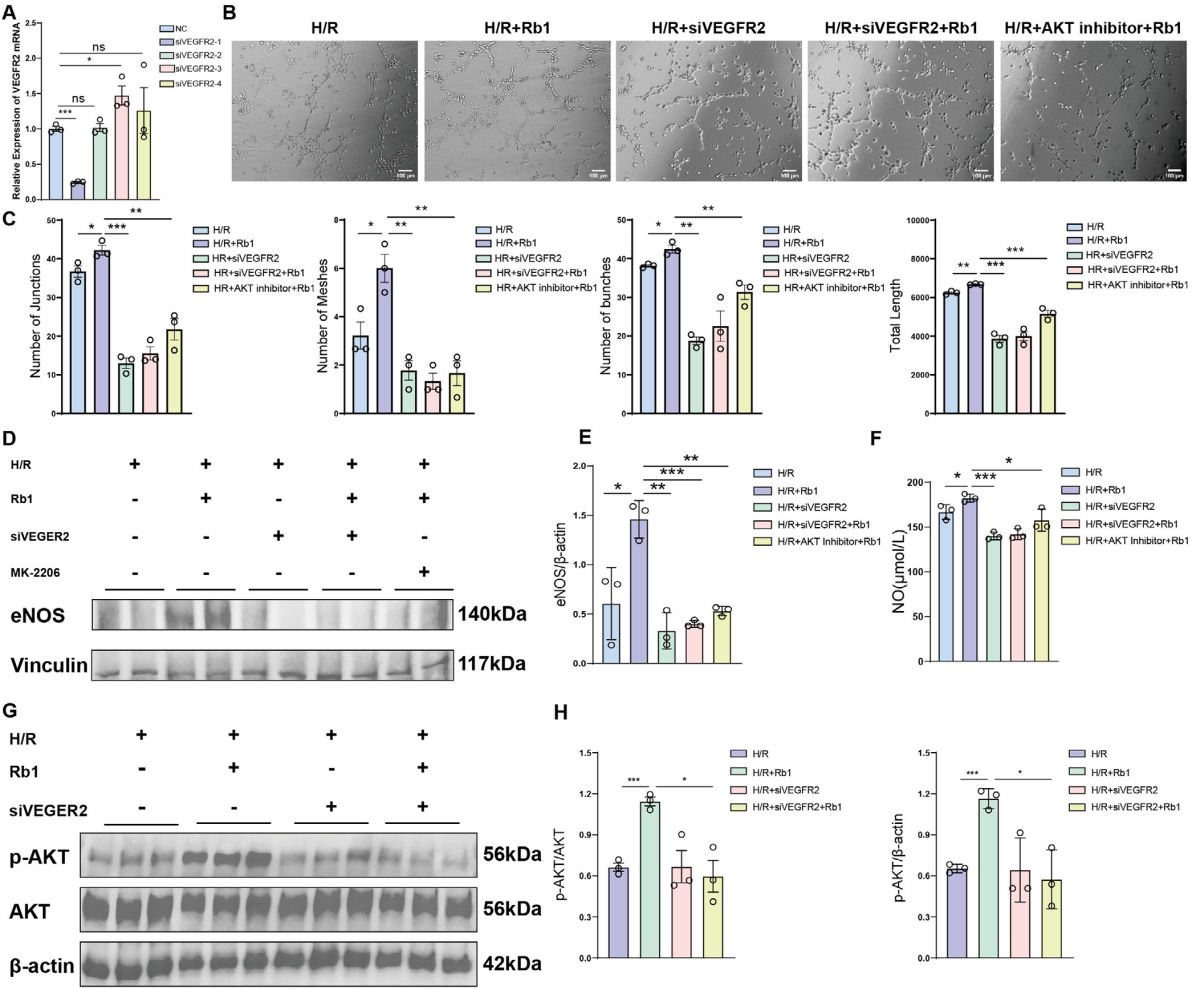
Inhibition of the VEGFR2/AKT signalling pathway reverses the therapeutic effects of Rb1. (A) The mRNA expression of VEGFR2. (B) Representative images of the effects of VEGFR2 knockdown and AKT inhibitor on HUVECs' tube formation ability. (C) The effects of VEGFR2 knockdown and AKT inhibitor on the formation of HUVEC tubular structure were quantitatively analysed. (D, E) Western blot images and the corresponding quantification of eNOS protein expression in HUVECs after VEGFR2 knockdown and AKT inhibitor addition. (F) The levels of NO secretion in HUVECs after the elimination of VEGFR2 and AKT inhibitors were detected for quantitative analysis. (G, H) Western blot images and the corresponding quantification of p‐AKT and AKT in HUVECs after VEGFR2 knockdown. Data are presented as the means ± SD. *n* = 3 pre groups of cells, ***p < 0.001, **p < 0.01, *p < 0.05 versus sham group. ***p < 0.001, **p < 0.01, *p < 0.05 versus uIRI group.

To block the binding of Rb1 to VEGFR2 in HUVECs, we used siRNA to knock down the expression of VEGFR2. After evaluating the transfection efficiency of four different siRNA, siVEGFR2‐1 was chosen for further study because of its highest efficiency (Figure 6A). We further investigated whether Rb1 promotes angiogenesis through the VEGFR2‐mediated AKT signalling pathway. VEGFR2 expression was knocked down in HUVECs using small interfering RNA (siRNA). The activation of the AKT signalling pathway was inhibited by adding the AKT inhibitor MK‐2206 (500 nM). Compared to Rb1‐pretreated HUVECs, weak tubular structures were observed in both the H/R + AKT inhibitor + Rb1 and the H/R + siVEGFR2 + Rb1 (Figure 6B,C). After adding Rb1 to stimulate HUVECs, knocking down VEGFR2 or blocking AKT signalling decreased the expression of eNOS and NO levels (Figure 6D–F). In addition, it was found that after VEGFR2 knockdown, p‐AKT expression was decreased, whereas that of total AKT remained unchanged (Figure 6G,H). These data suggest that the knockdown of VEGFR2 or the inhibition of the AKT signalling pathway weakens the therapeutic effect of Rb1 on promoting angiogenesis and improving vascular function.

## 4 Discussion

Renal fibrosis is a prevalent pathological characteristic and the ultimate manifestation of CKD. It is associated with endothelial injury and capillary rarefaction, yet there is a lack of efficacious drugs for its treatment. This study observed that Rb1 can effectively promote renal angiogenesis and alleviate renal fibrosis in mice transitioning from AKI to CKD by activating the AKT signalling pathway via targeting VEGFR2.

Abundant evidence has confirmed that Rb1 is therapeutic in multiple kidney diseases such as AKI, CKD and DKD and can improve kidney function and pathological injury [15, 40, 41]. The results suggest that Rb1 can significantly inhibit renal interstitial fibrosis in UUO rats, possibly through inhibiting oxidative damage and TGF‐β1 expression [42]. In addition, Rb1, recognised as a natural modulator of autophagy, can prevent autophagy activation in the kidneys of UUO mice and alleviate renal fibrosis in UUO mice [16]. Rb1 ameliorates EMT and renal fibrosis via inhibiting ROS production and activating the Bip/eIF2α/CHOP signalling pathway in HK2 cells [43]. Here, we also demonstrated that Rb1 exerts a renal protective effect in mice during the transition from AKI to CKD and can reduce renal fibrosis, the same as previously reported.

Capillary rarefaction and vascular endothelial injury are significant pathophysiological mechanisms that drive renal fibrosis and the transition from AKI to CKD [44, 45, 46, 47, 48]. The strategy of targeting vascular endothelial cells to restore renal microvessels might provide a novel therapeutic approach for delaying the transition from AKI to CKD. Rb1 has been reported to prevent homocystine‐induced dysfunction in endothelial progenitor cells via VEGF/p38MAPK and SDF‐1/CXCR4 activation [39]. Wu et al. [38] found that Rb1 incorporated silk/micro‐nano hydroxyapatite/sodium alginate composite promotes bone formation and angiogenesis. Interestingly, our findings suggest that Rb1 can enhance renal angiogenesis and attenuate renal fibrosis of uIRI‐induced mice. Furthermore, we confirmed that Rb1 promotes the tube formation of H/R‐induced HUVECs and protects the endothelial function.

Numerous studies have probed the binding targets of Rb1. It is reported that Rb1 can bind to NADH dehydrogenase within mitochondrial complex I, suppressing its activity in astrocytes [13]. The finding that Rb1 can bind to NADH dehydrogenase in mitochondrial complex I was also verified in the mice model of acute myocardial infarction [12]. Rb1 exhibits beneficial therapeutic effects in promoting renal angiogenesis and alleviating renal fibrosis, but the mechanism of this renal repair remains incompletely elucidated. Therefore, finding new ways to fully explain the proangiogenic mechanism of Rb1 in treating AKI to CKD transition is critical. We conducted virtual screening and molecular docking with VEGFR2 as the target and found that Rb1 was well combined with VEGFR2.

Within the VEGFR family, different subtypes (VEGFR1, VEGFR2 and VEGFR3) play differentiated roles in kidney diseases. VEGFR3 dominates lymphangiogenesis [49]. Among them, VEGFR1 and VEGFR2 are mainly distributed on the surface of vascular endothelium and regulate angiogenesis. Previous studies have shown that blocking VEGFA‐VEGFR2 signalling with soluble VEGFR1 can accelerate the progression of glomerulosclerosis, loss of PTCs, and interstitial fibrosis [50]. Therefore, we chose VEGFR2 as the main research object.

It has been reported that erythropoietin can alleviate LPS‐induced microvascular injury by increasing the expression of VEGFR2 in septic acute kidney injury models [51]. On the basis of prior research, our present study designates VEGFR2 as a crucial molecular target of Rb1 in promoting angiogenesis and clarifying its downstream signalling pathway. Our study demonstrated that Rb1 can up‐regulate p‐VEGFR2 expression in vivo and in vitro. The signal transduction pathways mediated by VEGFR2 mainly encompass the MAPK, PI3K/Akt, and FAK signal pathways [34]. Among them, AKT, a serine/threonine kinase, is one of the major downstream effector molecules of VEGFR2. The AKT signalling pathway modulates physiological processes, including cell proliferation, apoptosis, angiogenesis, and energy metabolism [52]. Zhou et al. [53] demonstrated that Rb1 activates the AKT signalling pathway to exert its function. Our results are consistent with those reported in the literature. Rb1 activates the AKT signalling pathway in uIRI mice and H/R‐induced HUVECs.

There were some limitations in this experiment. To begin with, the utilisation of VEGFR2 inhibitors and animal models with VEGFR2 suppressed expression can confirm the mechanism underlying Rb1's effects and ascertain its precise molecular target for binding. Additionally, we can use some experimental techniques, such as SPR and Pulldown, to confirm the combination of Rb1 and VEGFR2. The experiment could be further enhanced, and numerous mechanisms of Rb1 remain worthy of further exploration.

**FIGURE 7:**
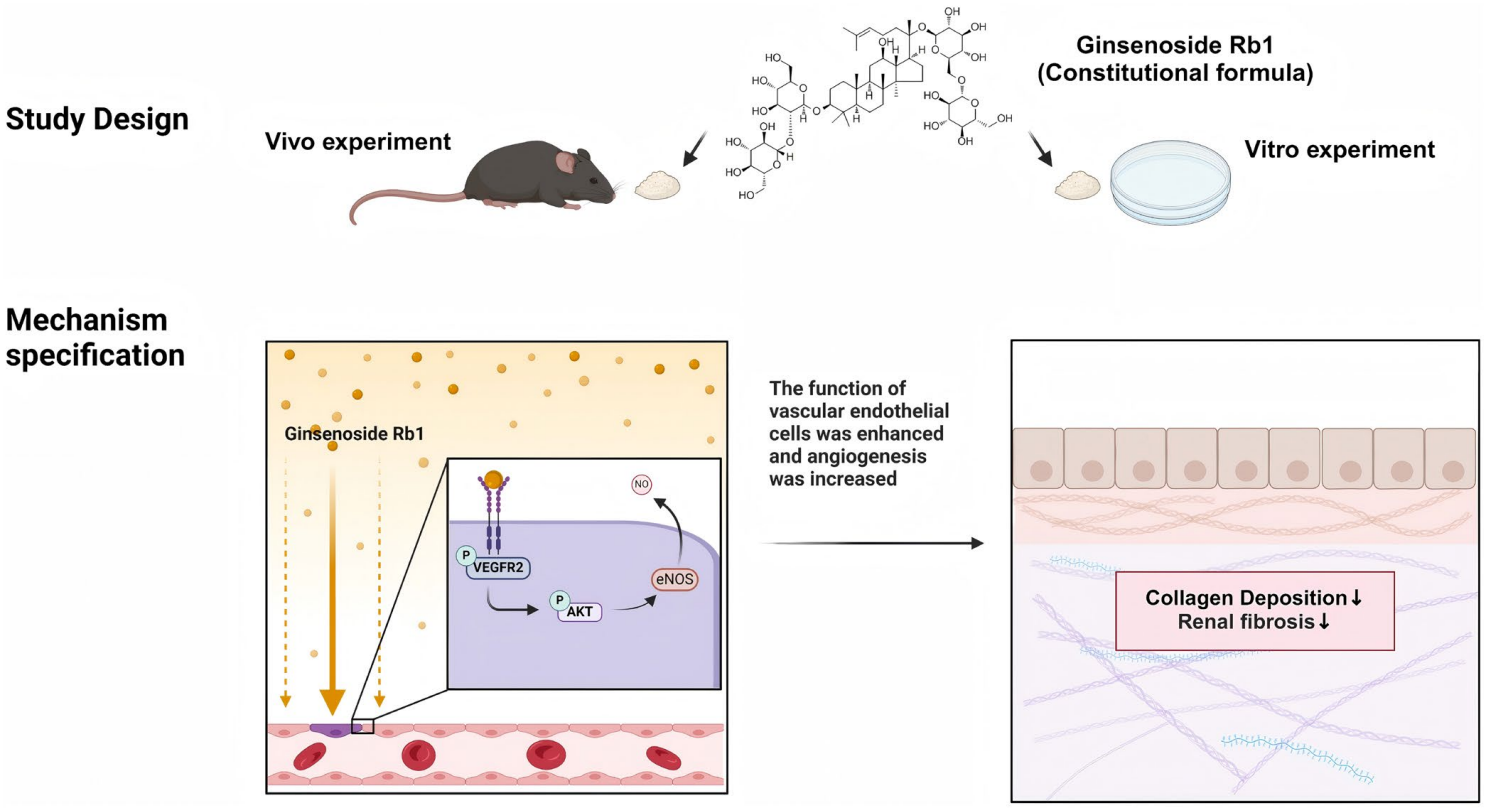
The mechanism of Rb1 in delaying the transition from AKI to CKD.

In summary, our study suggests that Rb1 can target VEGFR2, activate the AKT signalling pathway, and ultimately reduce kidney damage (Figure 7). Promoting renal angiogenesis may protect kidney function during AKI development. This study provides a potential treatment strategy for the transition from AKI to CKD and explores the interaction between Rb1 and VEGFR2.

## Author Contributions

**Keying Zhang:** conceptualization (equal), formal analysis (equal), methodology (equal), validation (equal), visualization (equal), writing – original draft (equal), writing – review and editing (equal). **Yuwei Ji:** writing – original draft (equal), writing – review and editing (equal). **Zhangning Fu:** software (equal), supervision (equal), validation (equal). **Xiaochen Wang:** data curation (equal), formal analysis (equal), investigation (equal). **Yifan Zhang:** software (equal), supervision (equal), validation (equal). **Yan Yang:** formal analysis (supporting), methodology (supporting), resources (supporting), software (supporting). **Ran Liu:** methodology (lead), project administration (lead), resources (lead). **Xiangmei Chen:** conceptualization (equal), data curation (equal), formal analysis (equal), funding acquisition (equal). **Guangyan Cai:** conceptualization (equal), funding acquisition (lead), validation (lead), writing – original draft (lead). **Quan Hong:** conceptualization (equal), data curation (equal), formal analysis (equal), funding acquisition (equal), project administration (equal), writing – review and editing (equal).

## Ethics Statement

All animal experimental procedures were approved by the Institutional Animal Care and Use Committee at the Chinese PLA General Hospital.

## Conflicts of Interest

The authors declare no conflicts of interest.

## Data Availability

The data supporting the findings of this research are available from the corresponding author upon request.
